# Treatment for femoroacetabular impingement: a qualitative method for exploring equipoise amongst hip arthroscopy surgeons

**DOI:** 10.1186/1745-6215-14-S1-P103

**Published:** 2013-11-29

**Authors:** Peter Wall, Alba Realpe, Damian Griffin, Rachel Hobson, Ann Adams

**Affiliations:** 1 Warwick Medical School, University of Warwick, Coventry, UK

## 

The published literature suggests uncertainty about whether operative or nonoperative treatments are best for femoroacetabular impingement (FAI). Without the same level of uncertainty (equipoise) amongst surgeons, a RCT will be challenging. A qualitative study was conducted to explore the level of equipoise amongst arthroscopic FAI surgeons. In phase 1, 14 hip arthroscopy surgeons were interviewed and asked to make treatment decisions based on real life cases that included actively recruiting patients to a theoretical RCT. In phase 2, 9 hip arthroscopy hip surgeons participating in a pilot RCT were interviewed about their experiences so far of taking part in a pilot RCT. Five surgeons took part in both phase 1 and 2. Sixteen (89%) surgeons believed that they were in equipoise and that a RCT was required to generate superior scientific evidence and guidelines for the care. Despite this 5 (36%) surgeons showed a lack of active clinical equipoise when faced with real life case scenarios or discussing involvement with a pilot RCT. Some of the reasons behind surgeons’ lack of equipoise, ranged from lack of belief in the FAI pathology, to personal enthusiasm and gut instinct about the efficacy of surgery on one hand; but conservatism on the other. Although many would like a RCT to guide care, there may be particular challenges amongst this same population when actively recruiting patients to a RCT. Qualitative methodology can be used to help design surgical RCTs and address any subsequent difficulties with recruitment.

